# Vav-iCre-Mediated Deletion of TFAM Is Not Recoverable and Is Consistent with Embryonic Lethality

**DOI:** 10.3390/genes17030255

**Published:** 2026-02-25

**Authors:** Rituparna Ghosh, Elina Shakur, Matthew J. Yousefzadeh

**Affiliations:** Columbia Center for Translational Immunology and Burch-Lodge Center for Human Longevity, Department of Medicine, Columbia University Medical Center, New York, NY 10032, USA; rg3652@cumc.columbia.edu (R.G.); eds2189@cumc.columbia.edu (E.S.)

**Keywords:** aging, genomic instability, immunology, immunosenescence, mitochondrial dysfunction

## Abstract

Genome stability is the cornerstone of cellular health, and imbalances can cause a number of outcomes, including aging, cancer, and other pathologies. DNA damage is a strong driver of both cellular senescence and mitochondrial dysfunction, two other key hallmarks of aging. Both nuclear and mitochondrial genome instability have been shown to drive aging in the hematopoietic system, which then propagates to non-lymphoid tissues, enhancing morbidity and mortality. The loss of TFAM, a key regulator of mitochondrial DNA replication and nucleoid stability, in T cells has been shown to cause mitochondrial dysfunction, leading to premature immune aging and eventual systemic aging. We sought to investigate whether the loss of TFAM in all immune cells would have a comparable or stronger effect on both the immune system and parenchyma. To address this, we attempted to generate *Vav-iCre^+/−^*; *Tfam^fl/fl^* mice, which are deficient in TFAM in all immune cells. However, this genotype was unrecoverable as no mutant pups were born, suggesting embryonic lethality. Conversely, we generated mice lacking SIRT6, a nuclear DNA repair enzyme that also regulates mitochondrial homeostasis, in all immune cells and found them to be viable and born at expected Mendelian frequencies. Our findings demonstrate the necessity of mitochondrial genome maintenance and homeostasis repair in immunity.

## 1. Introduction

Genome integrity is essential for cellular and physiological function; consequently, organisms have evolved robust repair pathways and responses to genotoxic insults [1]. Genomic instability can lead not only to transformation, which drives carcinogenesis, but also other cell fates such as apoptosis and cellular senescence [2]. A wealth of evidence from animal studies and human clinical studies demonstrates that DNA damage is a potent driver of the aging process [2,3,4]. In fact, genomic instability is one of the twelve hallmarks of aging, which represent key molecular perturbations that play a causal role in both chronological and biological aging [5,6]. Many in vitro and in vivo studies rely on treatments with supraphysiological doses of DNA-damaging agents or the deletion of critical DNA repair enzymes to instigate genome instability to investigate the impact on cellular function and physiological outcomes. While considerable focus is given to the damage and repair that occurs within the nuclear genome, the integrity of the mitochondrial genome is also critical for maintaining homeostasis [7,8,9].

Mitochondrial DNA (mtDNA) damage can accelerate biological aging by impairing the replication or transcription of indispensable genes involved in respiration and other processes, ultimately giving rise to mitochondrial dysfunction [10,11,12]. Unlike the nuclear genome, mtDNA integrity can be maintained through the degradation of damaged mitochondrial genomes via mitophagy [12,13]. mtDNA is organized into DNA–protein structures called nucleoids that reside within the mitochondrial matrix [14]. Each nucleoid typically contains a single mitochondrial genome bound by several proteins that regulate mitochondrial replication, transcription, repair, and packaging [15]. One of these proteins is Mitochondrial Transcription Factor A (TFAM), which is a key mitochondrial protein that is encoded within the nuclear genome. Beyond its role in transcription-primed mtDNA replication, the formation of mitochondrial DNA nucleoid structures, it also plays a role in fission-mediated mtDNA segregation [16,17,18,19]. The constitutive loss of *Tfam* is lethal; however haploinsufficiency (*Tfam^+/−^*) leads to enlarged nucleoids and mtDNA loss and leakage into the cytoplasm, which can stimulate cGAS-STING signaling [19,20]. The tissue-specific deletion of *Tfam* has been used to study the role of mitochondrial dysfunction in aging and age-related chronic diseases [21,22,23,24,25,26,27]. Notably, the deletion of *Tfam* in T cells causes *CD4-Cre^+/−^*; *Tfam^fl/fl^* mice to rapidly develop numerous features of immunosenescence [24], the progressive age-related decline in immune function [28,29]. Furthermore, *CD4-Cre^+/−^*; *Tfam^fl/fl^* mice eventually develop premature aging in non-lymphoid organs well after the onset of immunosenescence. This phenomenon is driven by both cell autonomous and cell non-autonomous effects, which result in premature senescence in the solid organs that shorten both the healthspan and lifespan in *CD4-Cre^+/−^*; *Tfam^fl/fl^* mice [24]. Even the other immune cell lineages can be affected by the T-cell-specific loss of *Tfam*. For example, the remodeling of the bone marrow niche and enhanced granulopoiesis, which contributed to age-related phenotypes, were observed in *CD4-Cre^+/−^*; *Tfam^fl/fl^* mice [27].

Previously, it has also been shown that the loss of the DNA repair gene *Ercc1* in hematopoietic cells of mice (*Vav-iCre^+/−^*; *Ercc1^−/fl^* mice), disrupting multiple DNA repair pathways and leading to the accumulation of spontaneously occurring endogenous DNA damage, caused similar effects [30]. This led to interest in whether the effect of mitochondrial dysfunction broadly across all immune cells (via loss of *Tfam*) would yield a similar or stronger phenotype than that observed in *CD4-Cre^+/−^*; *Tfam^fl/fl^* mice [24]. Thus, *Tfam* conditional floxed mice were crossed with *Vav-iCre* mice that express a codon-improved variant of the cre recombinase (iCre) expressed under the control of the murine *vav1* oncogene promoter that induces recombination in hematopoietic stem cells and their progenitors [31]. While useful for studying immune biology, *Vav-iCre* mice are not without limitations, such as potential leaky expression in non-target tissues (further discussed in the Results and Conclusions Sections) [32]. We hypothesized that *Vav-iCre^+/−^*; *Tfam^fl/fl^* mice would have an accelerated or stronger phenotype than *CD4-Cre^+/−^*; *Tfam^fl/fl^* mice [24], but we were surprised at the inability to recover a *Vav-iCre^+/−^*; *Tfam^fl/fl^* mouse from multiple breedings. To further investigate the role of DNA repair deficits in murine immunology, we deleted the DNA repair factor SIRT6, which is localized to the nucleus but plays a role in regulating mitochondrial homeostasis [33,34,35], in all murine immune cells (*Vav-iCre^+/−^*; *Sirt6^fl/fl^* mice). Interestingly, *Vav-iCre^+/−^*; *Sirt6^fl/fl^* mice are viable and born at near Mendelian frequencies. These results highlight the importance of TFAM and mtDNA stability in homeostasis and immune function.

## 2. Methods

### 2.1. Animal Studies

All animal studies were approved by the Columbia University Institutional Animal Care and Use Committee in accordance with institutional, local, state, and federal regulations. All animal breeding studies were carried out from May 2023 to November 2025. *Tfam^fl/fl^* (#026123), *Sirt6^fl/fl^*, (#017334), *CD4-Cre^+/−^* (#017336), and *Vav-iCre^+/−^* (#008610) mouse strains were all obtained from The Jackson Laboratory (Bar Harbor, ME) and maintained on a C57BL/6J background. All mice were housed in a specific pathogen-free condition barrier facility with routine serological analysis of sentinel mouse cages. *Sirt6^fl/fl^* mice were originally provided on a mixed background and backcrossed onto the C57BL/6J strain using speed congenics, and genetic background was confirmed via genetic monitoring services provided by TransnetYX. All immune-specific mutant mice (*X-Cre^+/−^*; *Y^fl/fl^*; X = *CD4-Cre* or *Vav-iCre*; Y = *Sirt6*, or *Tfam*) used in the experimental cohorts were generated by crossing homozygous floxed (*Sirt6^fl/fl^*, *Tfam^fl/fl^*) mice with the individual compound heterozygous cre mice (*X-Cre^+/−^*; *Y^+/fl^*; X = *CD4-Cre* or *Vav-iCre*; Y = *Sirt6*, or *Tfam*) with the same genetic background (C57BL/6J). For *Vav-iCre* breedings, only females carrying the *Vav-iCre* allele were used for breeding to limit potential germline leakage that has been previously reported in male *Vav-iCre* mice. All offspring from crosses were given ear tags with unique identifiers, and ear punches were used for genotyping services provided by TransnetYX. Post-natal mortality of pups was monitored daily by animal laboratory technicians.

### 2.2. Immunoblotting

Tissues were isolated from two-month-old mice, and protein lysates were produced using T-PER buffer (Thermo-Fisher, Waltham, MA, USA) according to manufacturer’s specification. Polyacrylamide (4–12%) gel electrophoresis was performed before protein was transferred to PVDF membrane and blocked for 1 h in 10% milk TBST at room temperature. Membranes were incubated overnight at 4 °C in primary antibodies: anti-SIRT6 (Cell Signaling Technology, Danvers, MA, USA, catalog #12486, 1:500 dilution in TBST) and anti-Vinculin (Abcam, Waltham, MA, USA, catalog #ab129002, 1:2000 dilution in TBST). Following three 5 min washes in TBST, membranes were incubated for 2 h at room temperature with a goat anti-rabbit HRP-conjugated secondary antibody (Millipore Sigma, Burlington, MA, USA, catalog #A0545, 1:2000 dilution in TBST). Membranes were then washed three additional times for 5 min in TBST, and protein detection was performed by chemiluminescence detection using ECL reagent (Pierce, Rockford, IL, USA).

### 2.3. Statistical Analysis

Genotyping data (expected versus observed) was plotted in GraphPad Prism version 10.6.0, and statistical analysis was performed using Chi-squared (χ^2^) analysis.

## 3. Results

**Figure 1:**
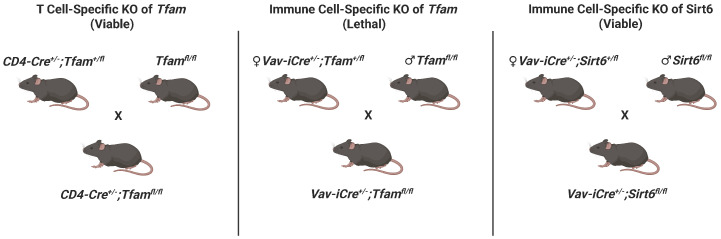
Breeding schematic for crosses. Individual breeding schemes show crosses to generate *CD4-Cre^+/−^*; *Tfam^fl/fl^*, *Vav-iCre^+/−^*; *Tfam^fl/fl^*, and *Vav-iCre^+/−^*; *Sirt6^fl/fl^* mutant mice and their status (lethal/viable). Please note that *Vav-iCre* breeding schemes assign cre alleles to female mice to reduce potential off-target germline effects. The figure was created with BioRender.

**Figure 2:**
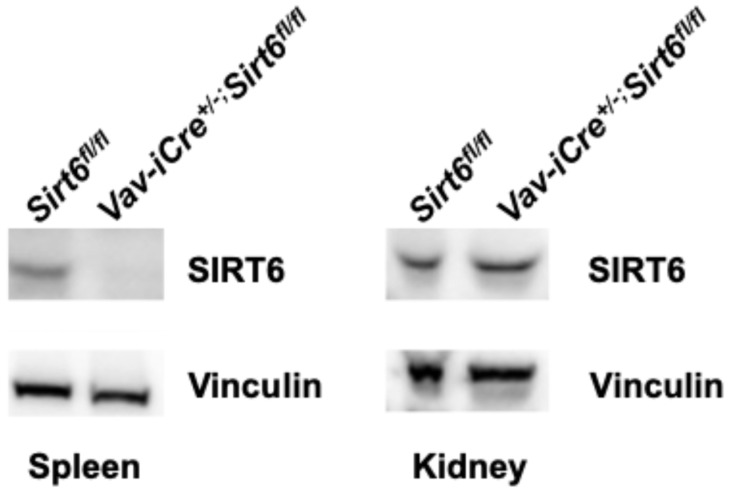
Targeted loss of SIRT6 in the spleens of *Vav-iCre^+/−^*; *Sirt6^fl/fl^* mice. Immunoblots analysis of spleen and kidney tissues from two-month-old *Vav-iCre^+/−^*; *Sirt6^fl/fl^* mice and *Sirt6^fl/fl^* control mice, assessing SIRT6 and Vinculin protein abundance.

In order to study the impact of loss of TFAM across immune cells, we sought to generate *Vav-iCre^+/−^*; *Tfam^fl/fl^* mice. Because of prior reports of aberrant cre recombinase expression in the testis of Vav-iCre mice, only female Vav-iCre breeders were used in mating pairs (Figure 1, Table S1). Unfortunately, after examining the offspring in several litters (n = 26) resulting from crosses of female *Vav-iCre^+/−^*; *Tfam^+/fl^* mice with male *Tfam^fl/fl^* breeders, no *Vav-iCre^+/−^*; *Tfam^fl/fl^* mice were present despite an expectation of a 25% frequency (0 observed versus ~41 expected *Vav-iCre^+/−^*; *Tfam^fl/fl^* offspring; *p* < 0.001) (Table 1). All other relevant genotypes (*Vav-iCre^+/−^*; *Tfam^+/fl^*, *Tfam^+/fl^*, *Tfam^fl/fl^* mice) were born at near Mendelian frequencies. Furthermore, we observed no post-natal lethality in pups from the litters, suggesting embryonic lethality. This stands in stark contrast to *CD4-Cre^+/−^*; *Tfam^fl/fl^* mice, which are viable and born at expected frequencies (25 observed versus ~26 expected *CD4-Cre^+/−^*; *Tfam^fl/fl^* offspring) (Table 1). Meanwhile, a similar breeding strategy was employed to create *Vav-iCre^+/−^*; *Sirt6^fl/fl^* mice to limit off-target effects (Figure 1). *Vav-iCre^+/−^*; *Sirt6^fl/fl^* mice were born at a ~30.7% frequency across multiple litters (20 observed versus ~16 expected *Vav-iCre^+/−^*; *Sirt6^fl/fl^* offspring) (Table 1). Likewise, no post-natal mortality was observed in pups from the *Vav-iCre*; *Sirt6* breedings. An immunoblot analysis of spleens from *Vav-iCre^+/−^*; *Sirt6^fl/fl^* mice confirmed a loss of SIRT6, whereas the renal levels of SIRT6 protein in the kidneys of these mice were comparable to those in the *Sirt6^fl/fl^* control mice (Figure 2).

## 4. Discussion

Prior studies demonstrated that T-cell-specific knockouts of *Tfam* developed multiple features of immunosenescence before the development of secondary senescence in peripheral tissues [24]. *CD4-Cre^+/−^*; *Tfam^fl/fl^* mice develop multimorbidity as a result of both the loss of protective immune functions and the adoption of pathogenic immune changes [36]. Similar changes were discovered in *Vav-iCre^+/−^*; *Ercc1^−/fl^* mice, which lack *Ercc1* in multiple immune lineages [30]. This led to the investigation of the broad deletion of *Tfam* in all immune cells rather than just T cells as the rationale for generating *Vav-iCre^+/−^*; *Tfam^fl/fl^* mice. While other immune-specific deletions of *Tfam* are viable [24,37,38,39,40], our findings raise issue with deleting *Tfam* throughout the immune system (*Vav-iCre^+/−^*; *Tfam^fl/fl^* mice). Our inability to generate this mouse suggests embryonic lethality, although this possibility requires further investigation. Together with prior reports, our findings underscore the importance of mtDNA homeostasis in the immune system. Furthermore, there is a paucity of studies related to the immune-specific deletion of mitochondrial maintenance genes let alone mtDNA repair genes, thus leaving much biology to be explored. Future investigations into the lethality of *Vav-iCre^+/−^*; *Tfam^fl/fl^* mice should make use of timed pregnancies to determine on which day of gestation embryonic lethality occurs and if this coincides with key developments of fetal hematopoiesis.

While useful, *Vav-iCre* mice are less than ideal due to reports of cre expression in testis that do not express *Vav*, potentially due to transgene insertion effects [31,41,42]. Beyond this, another study crossed a stop-floxed-enhanced Yellow Fluorescent Protein reporter into *Vav-iCre^+/−^* mice (*Vav-iCre^+/−^*; *Rosa26^+/LSL-EYFP^*) and found recombination (EYFP^+^ cells) both in hematopoietic stem cells and in Lin^−^ CD45^−^ CD31^−^ CD51^−^ Sca-1^−^ bone cells that lack hematopoietic surface markers [32]. Previously, studies showed that *Vav-iCre^+/−^*; *Ercc1^−/fl^* mice demonstrated a loss of *Ercc1* in lymphoid tissues but not in parenchyma tissues, such as the aorta, kidney, or liver [30]. Our immunoblotting data from *Vav-iCre^+/−^*; *Sirt6^fl/fl^* mice yielded no evidence of an off-target loss of SIRT6 in the kidneys. While this data argues against significant “leaky” germline cre expression in *Vav-iCre^+/−^*; *Sirt6^fl/fl^* mice, more comprehensive, multi-tissue analyses are necessary to definitively exclude this possibility.

The use of other broader immune-specific cre lines—like *hCD2-iCre,* which drives the cre recombinase from the human *CD2* promoter—may be a useful alternative [32,43]. However, their expression is limited to the lymphocyte lineage and not myeloid cells and still suffers from potential off-target expression in the testis [32]. Some of these limitations to potentially targeting *Tfam* and other mitochondrial maintenance genes across a wide swath of immune cells could be overcome by the development of more precise hematopoietic stem-cell-specific cre lines or the use of inducible cre (ex. tamoxifen-inducible CreERT2 models) that could be employed post-development in mature mice. Even if some off-target cre expression was still present, the reconstitution of immune systems in recipient mice after immunoablation with hematopoietic stem cells from donor *Tfam* immune-deficient mice could be employed as an alternative. These findings highlight the critical nature of mitochondrial genome integrity in the immune system and the need for better tools to study the immune-wide deletion of DNA repair genes to better understand their role in immune function and limiting immunosenescence.

## Figures and Tables

**Table 1:** Mendelian frequency of immune-specific mutants. Statistical significance determined using Chi-squared analysis. *** *p* < 0.001; ns = not significant.

Genotype	Litters (Offspring)	Expected #	Observed #	Frequency	Significance
*CD4-Cre^+/−^*; *Tfam^fl/fl^*	17 (103)	~26	25	24.3%	ns
*Vav-iCre^+/−^*; *Tfam^fl/fl^*	26 (166)	~42	0	0.00%	***
*Vav-iCre^+/−^*; *Sirt6^fl/fl^*	10 (65)	~16	20	30.7%	ns

## Data Availability

The original contributions presented in this study are included in the article/Supplementary Material. Further inquiries can be directed to the corresponding author.

## Supplementary Materials

The following supporting information can be downloaded at: https://www.mdpi.com/article/10.3390/genes17030255/s1, Table S1: Breeding details.
