# Supplementary material for: Vav-iCre-Mediated Deletion of TFAM Is Not Recoverable and Is Consistent with Embryonic Lethality
Source: Genes (Basel). 2026 Feb 25;17(3):255. doi: 10.3390/genes17030255 (PMC13025505; doi:10.3390/genes17030255)
Supplement: Supplementary file 1 [file genes-17-00255-s001.zip › genes-4092091-supplementary.pdf]

Vav-iCre;Tfam		
**Litters**		26
**Offspring**		166
**Genotype**	**Observed**	
Tfamfl/fl		54
Tfam+/fl		52
Vav-iCre+/-;Tfam+/fl		60
Vav-iCre+/-;Tfamfl/fl		0

CD4-Cre;Tfam		
**Litters**		17
**Offspring**		103
**Genotype**	**Observed**	
Tfamfl/fl		30
Tfam+/fl		27
CD4-Cre+/-;Tfam+/fl		21
CD4-Cre+/-;Tfamfl/fl		25

Vav-iCre;Sirt6		
**Litters**		10
**Offspring**		65
**Genotype**	**Observed**	
Sirt6fl/fl		13
Sirt6+/fl		15
Vav-iCre+/-;Sirt6+/fl		17
Vav-iCre+/-;Sirt6fl/fl		20
